# Natural selection contributes to geographic patterns of thermal plasticity in *Plantago lanceolata*

**DOI:** 10.1002/ece3.4977

**Published:** 2019-02-14

**Authors:** Matthew M. Marshall, Leslie C. Batten, David L. Remington, Elizabeth P. Lacey

**Affiliations:** ^1^ Department of Biology University of North Carolina at Greensboro Greensboro North Carolina

**Keywords:** genetic drift, geographic clines, natural selection, phenotypic plasticity, temperature, thermal plasticity

## Abstract

A long‐standing debate in evolutionary biology concerns the relative importance of different evolutionary forces in explaining phenotypic diversification at large geographic scales. For example, natural selection is typically assumed to underlie divergence along environmental gradients. However, neutral evolutionary processes can produce similar patterns. We collected molecular genetic data from 14 European populations of *Plantago lanceolata* to test the contributions of natural selection versus neutral evolution to population divergence in temperature‐sensitive phenotypic plasticity of floral reflectance. In *P*. *lanceolata,* reflectance plasticity is positively correlated with latitude/altitude. We used population pairwise comparisons between neutral genetic differentiation (*F*_ST_ and Jost's *D*) and phenotypic differentiation (*P*_ST_) to assess the contributions of geographic distance and environmental parameters of the reproductive season in driving population divergence. Data are consistent with selection having shaped large‐scale geographic patterns in thermal plasticity. The aggregate pattern of *P*_ST_ versus *F*_ST_ was consistent with divergent selection. *F*_ST_ explained thermal plasticity differences only when geographic distance was not included in the model. Differences in the extent of cool reproductive season temperatures, and not overall temperature variation, explained plasticity differences independent of distance. Results are consistent with the hypothesis that thermal plasticity is adaptive where growing seasons are shorter and cooler, that is, at high latitude/altitude.

## 1 INTRODUCTION

A long‐standing debate in evolutionary biology is the relative importance of different evolutionary forces in explaining phenotypic diversification (e.g., Hangartner, Laurila, & Räsänen, 2012; Koskinen, Haugen, & Primmer, 2002; Leinonen, O'Hara, Cano, & Merilä, 2008; O'Hara & Merilä, 2005). For example, natural selection is typically assumed to explain population divergence along environmental gradients. However, neutral processes, for example, genetic drift with limited gene flow, can also produce divergence, particularly in populations with small effective population sizes, *N*_e_ (Lande, 1992; Wright, 1931). Currently, there is a need to assess the relative importance of selection versus neutral processes at large geographic scales (Bragg, Supple, Andrew, & Borevitz, 2015; Kawakami et al., 2011; Kleynhans, Mitchell, Conlong, & Terblanche, 2014; Michalski, Malyshev, & Kreyling, 2017; Orsini, Vanoverbeke, Swillen, Mergeay, & Meester, 2013; Walisch, Colling, Bodenseh, & Matthies, 2015). Such information not only helps us to understand how evolutionary processes have molded current geographic patterns of phenotypic diversification, the information also helps us better predict how climate change will alter current geographic patterns. Here, we provide what we believe is the first study to examine whether these processes contribute to population divergence in thermal plasticity, that is, phenotypic plasticity in response to temperature, along large‐scale latitudinal and altitudinal gradients.

Thermal plasticity displays a positive correlation with latitude and/or altitude in many traits of diverse taxa, particularly in ectotherms (Angilletta, 2009; Ghalambor, Huey, Martin, Tewksbury, & Wang, 2006; Hoffmann, Sørensen, & Loeschcke, 2003). For example, clinal variation of temperature‐sensitive traits occurs in the developmental rate of frogs (Laugen, Laurila, Räsänen, & Merilä, 2003), thermal tolerance of insects (Addo‐Bediako, Chown, & Gaston, 2000; Gaston & Chown, 1999) and lizards (van Berkum, 1988), leaf shape in trees (Royer, Meyerson, Robertson, & Adams, 2009) and floral color and reflectance in plants (Lacey, Lovin, Richter, & Herington, 2010). While natural selection is generally assumed to have produced these clinal patterns, demographic history (i.e., past and current migration, founder effects, and genetic drift) may also have contributed to the phenotypic clines (Alho et al., 2010; Hancock et al., 2011; Hangartner et al., 2012; Kawakami et al., 2011; Kleynhans et al., 2014; Luquet, Léna, Miaud, & Plénet, 2015; Molina‐Montenegro & Naya, 2012; Montesinos‐Navarro, Picó, & Tonsor, 2012; Muir, Biek, Thomas, & Mable, 2014; Nadeau, Meirmans, Aitken, Ritland, & Isabel, 2016). Thus, although the clinal patterns are common, their causes remain largely untested.

Teasing apart the contributions of neutral and non‐neutral evolutionary forces in population divergence remains a challenge because both can produce similar geographic patterns (Huey, Gilchrist, Carlson, Berrigan, & Serra, 2000; Nadeau et al., 2016; Orsini et al., 2013; Whitlock, 2008). For example, founder effects such as migration out of refugia, coupled with limited gene flow and genetic drift, can produce a pattern of isolation by distance, that is, a positive correlation between genetic and phenotypic differentiation, and geographic distance between populations (IBD, Hutchison & Templeton, 1999; Wright, 1943). However, this geographic pattern can also be produced by local adaptation when the environmental conditions driving selection against nonlocally adapted migrants are correlated with distance, resulting in isolation by environment/adaptation (IBE/A, Hendry, 2004; Nosil, Vines, & Funk, 2005; Nosil, Funk, & Ortiz‐Barrientos, 2009; Orsini et al., 2013). In such cases, sampling the landscape in such a way that reduces the spatial–environment correlation may help disentangle the contributions of these different evolutionary forces.

**Figure 1:**
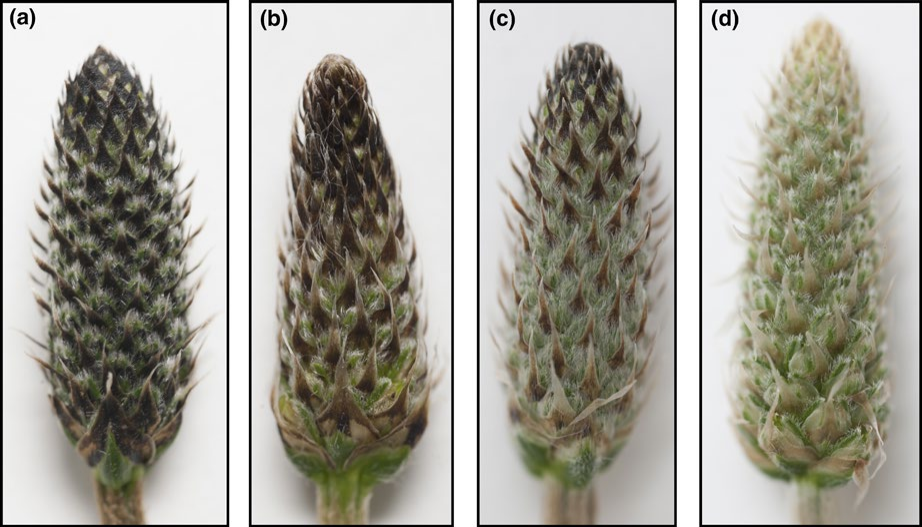
Under cool growing conditions (e.g., 15°C day/10°C night), *Plantago lanceolata *individuals vary in their ability to produce dark preflowering spikes (inflorescences of tightly packed flowers prior to stigma emergence) that exhibit low reflectance. When growing conditions are warm (e.g., 27°C day/20°C night), nearly all individuals produce spikes that are lightly colored and highly reflective (as in D). As the color of spikes lighten from left to right (a–d), the percentage of light reflected at 850 nm (i.e., floral reflectance) increases

The natural geographic variation in thermal plasticity of floral reflectance among populations of *Plantago lanceolata*, a widespread temperate herb, allowed us to address these challenges. In this species, many individuals can modify the color and NIR (near infrared) reflectance of a spike (i.e., an inflorescence of tightly packed flowers) in response to the ambient temperature experienced during flower development (Lacey & Herr, 2005). Typically, nonplastic individuals produce lightly colored/highly reflective spikes, regardless of ambient temperature (Lacey & Herr, 2005). In contrast, plastic individuals produce darker, less reflective spikes in cool temperatures, and lighter, more reflective spikes in warm temperatures (Figure 1; Lacey & Herr, 2005; Lacey et al., 2010). The darker, less reflective spikes can absorb more incoming solar radiation than lighter, more reflective spikes, thereby helping to warm reproductive tissues (Lacey & Herr, 2005). Conversely, during warm reproductive periods, lighter, high reflectance spikes absorb less incoming solar radiation, helping to cool tissues (Lacey & Herr, 2005). Thus, the darker, less reflective spikes appear to be beneficial in cool environments where warming tissues are advantageous (Lacey et al., 2010). Manipulative experiments provide evidence that this plasticity improves fitness via increased reproductive success during cool periods of the reproductive season, but does not negatively affect fitness during warm periods (Lacey, Lovin, & Richter, 2012). Furthermore, warming reproductive tissues during cool periods can enhance offspring fitness (Lacey, 1996; Lacey & Herr, 2000). At this point, no cost of this type of plasticity has been detected in terms of reproductive output (Lacey et al., 2012).

This temperature‐sensitive floral reflectance plasticity naturally varies along latitudinal and altitudinal gradients (Lacey et al., 2010). As latitude or altitude increases, the degree of temperature sensitivity and the proportion of plastic individuals in a population both increase, while the proportion of nonplastic individuals, which constitutively produce light colored/highly reflective flowering spikes, decreases (Lacey et al., 2010). Nonplastic individuals constitutively producing dark/poorly reflective spikes have rarely been found (Lacey et al., 2010).

To assess the evolutionary cause(s) of variation in floral reflectance plasticity in *P. lanceolata,* we compared neutral genetic differentiation (*F*_ST_ and Jost's *D*), phenotypic differentiation (*P*_ST_), geographic distance, and four parameters of the reproductive season environment. *F*_ST_ versus *Q*_ST_ or *P*_ST_ comparisons are an important tool for inferring the relative importance of neutral evolution versus natural selection (Hangartner et al., 2012; Leinonen et al., 2008; Whitlock, 2008). *F*_ST_ estimates how much population differentiation can be explained in the absence of natural selection; that is, it provides a null hypothesis to that of natural selection. The comparison can be used to determine whether populations have diverged for a trait, and to determine whether a series of populations along an environmental gradient show evidence of local adaptation. Among each pair of populations, the comparison produces one of three possible outcomes: *F*_ST_ = *Q*_ST_ or *P*_ST_, *F*_ST_ > *Q*_ST_ or *P*_ST_, or *F*_ST_ < *Q*_ST_ or *P*_ST_ indicating observed differences are best explained by neutral genetic drift, stabilizing selection, or diversifying selection, respectively (McKay & Latta, 2002; Merilä & Crnokrak, 2001).

The goals for our study were to collect and analyze molecular genetic AFLP data in order to (a) estimate neutral genetic population diversity and differentiation (*F*_ST_) for 14 populations of *P. lanceolata* in their native European environment, (b) infer patterns of migration and admixture potentially shaping population differentiation, (c) test whether isolation by distance explained a significant proportion of the variance in neutral AFLP markers, (d) compare *F*_ST_ and *P*_ST_ to test for a signature of natural selection, and (e) assess whether phenotypic divergence in thermal plasticity between populations is best explained by distance between populations, neutral evolution, and/or specific environmental properties of the reproductive season that might drive population divergence. The environmental variables we examined allowed us to evaluate potential drivers of selection. We used Mantel tests to examine whether geographic distance and neutral genetic differentiation (*F*_ST_ or Jost's *D*) were correlated, and whether geographic distance and population differences in each environmental variable were correlated. We used multiple regression of distance matrices (MRM) analyses to determine whether variation in *P*_ST_ could be explained by *F*_ST_ and Jost's *D*, geographic distance, and/or environmental properties of the reproductive season. We used linear regression models to examine how phenotypic and genetic differentiation varied along axes of geographic distance and environmental differences.

Additionally, our dataset allowed us to test two local adaptation hypotheses. The local adaptation hypothesis predicts that phenotypic properties of different populations should diverge as selective pressures become more different, and should do so at a greater rate than neutral genetic differences (Orsini et al., 2013). The Thermal Magnitude Hypothesis, which we will refer to as the Magnitude Hypothesis, and the Relative Frequency Hypothesis, which we will refer to as the Frequency Hypothesis, attempt to explain why exhibiting thermal plasticity is more adaptive at higher latitudes and altitudes than being nonplastic. The Magnitude Hypothesis states that thermal plasticity is more adaptive at higher latitudes and altitudes *because the magnitude of thermal variation is greater in these regions* (Ghalambor et al., 2006; Janzen, 1967). The Magnitude Hypothesis predicts phenotypic differentiation will correlate with environmental differences in the magnitude of thermal variation (i.e., temperature range) among populations. The Frequency Hypothesis states that thermal plasticity is more adaptive at higher latitudes and altitudes *because thermally variable growing seasons are shorter and individuals experience a relatively greater proportion of their growing season at cool, rather than warm temperatures* (Lacey et al., 2010). The Frequency Hypothesis predicts phenotypic differentiation will correlate with environmental differences in season duration and the proportion of cool temperatures during the growing season among populations. Lacey et al. (2010) have shown that phenotypic patterns of thermal plasticity among European populations of *P. lanceolata *are consistent with the Frequency Hypothesis and not the Magnitude Hypothesis. We examined whether their conclusions were supported when we compare the phenotypic patterns with genetic patterns generated by our AFLP data. Finally, our results allowed us to predict how climate change is likely to alter latitudinal and altitudinal patterns of thermal plasticity via thermal change affecting the intensity of natural selection.

## 2 METHODS

### 2.1 Biology of *P. lanceolata*

*Plantago lanceolata* L. (ribwort plantain, English plantain) is a temperate perennial herb native to Eurasia. A weedy species, it is an obligate outcrosser that is primarily wind‐pollinated. Reflectance (i.e., the amount of light reflected) and the color of a spike (i.e., an inflorescence of tightly packed flowers) are thermally plastic and are determined by the ambient temperature experienced during flower development (Lacey & Herr, 2005). For a single flower on a spike, floral reflectance becomes fixed at the time of flower development. However, floral reflectance is reversible for an individual plant. An individual plant typically produces spikes throughout an often‐lengthy flowering season, for example, 2–6 months depending on location. Also, the external temperature changes during that time. These changes induce the individual plant to modify the floral reflectance of its newly produced spikes. Floral reflectance thermal plasticity is strongest in the visible and NIR regions of the electromagnetic spectrum (Lacey & Herr, 2005).

Floral reflectance plasticity is genetically variable within and among natural populations of *P. lanceolata* and is positively correlated with latitude and altitude (Lacey & Herr, 2005; Lacey et al., 2010; Umbach, Lacey, & Richter, 2009). Individuals from low latitudes and altitudes often display negligible thermal plasticity and produce only highly reflective/lightly colored spikes. Most individuals from higher latitudes and altitudes reduce reflectance/darken spikes in response to cool environments, but do so to different degrees.

### 2.2 Experimental populations

We selected fourteen European *P. lanceolata* populations of the 29 used in Lacey et al. (2010). Populations spanned a latitudinal range of 39.3–50.9°N and an altitudinal range of 1–1,886 m (Table 1). When compared to the larger pool of 29 populations from Lacey et al. (2010), the populations we sampled spanned the southern 53% of latitudinal range, covered all of the altitudinal range, and sampled the lower 78% of the total variation in floral reflectance plasticity. Distance between populations was determined by uploading latitude–longitude coordinates into Google Earth (earth.google.com) as (a) minimum linear Euclidean distance in meters and (b) minimum geographic distance over land as determined using the path tool. Analyses conducted with Euclidean distance and distance over land produced the same conclusions; those with distance over land are presented because they are the most biologically reasonable.

**Table 1:** Population locations and characteristics: Country of origin, location within country, population symbol, mean heterozygosity, mean floral reflectance plasticity, and the number of individuals measured (*N*)

Source country	Location in country	Symbol	Latitude (°N)	Longitude (°E)	Altitude (m)	Mean Heterozygosity (±2*SD*); *N*	Mean reflectance plasticity (±2*SD*); *N*
France	Massif de la Chartreuse	FrG	45.37	5.4	1,000	0.266 (0.01); 19	28.005 (29.30); 25
	Hameau de St. Felix	FrH	43.58	3.97	35	0.267 (0.01); 23	19.346 (24.45); 27
	St. Pierre, Ile d'Oléron	FrI	45.95	−1.29	10	0.284 (0.01); 21	25.878 (29.66); 22
	St. Martin d'Hére	FrM	45.17	5.77	230	0.239 (0.01); 29	27.857 (22.45); 26
	St. Martin d'Uriage	FrMu	45.15	5.83	684	0.274 (0.01); 14	22.045 (30.67); 13
	Orsay	FrO	48.68	2.18	80	0.279 (0.01); 12	27.687 (32.19); 17
	L'Alpe d'Huez	FrR	45.09	6.07	1,886	0.249 (0.01); 34	27.103 (32.27); 26
Germany	Jena	GJ	50.93	11.58	150	0.254 (0.01); 30	17.011 (22.26); 30
Italy	Aprilia	IA	41.6	12.65	70	0.265 (0.01); 20	7.652 (14.75); 16
	Bagni di Vinadio	IB	44.3	7.08	1,300	0.297 (0.01); 27	26.231 (26.32); 23
	Castel Volturno	ICa	41.03	13.93	1	0.268 (0.01); 33	10.802 (17.86); 29
	Cosenza	ICs	39.3	16.25	238	0.280 (0.01); 10	11.671 (20.82); 7
Spain	Cangoria	SpC	42.69	−0.52	1,080	0.265 (0.01); 22	15.138 (23.18); 24
	Orbil de Villanua	SpO	42.66	−0.54	920	0.265 (0.01); 21	25.822 (31.23); 24

The phenotypic data for thermal plasticity of the genotypes in our sample populations came from an earlier study (Lacey et al., 2010). In that study, seeds collected in the year 2000 from each population were, grown, and passed through one generation in a common greenhouse environment, followed by within‐population pollination in a common growth chamber environment. Parental temperature effects were largely reduced by controlling the postzygotic temperature during flower development and seed maturation (Case, Lacey, & Hopkins, 1996; Lacey & Herr, 2000). Second‐generation individuals (also distinct genotypes) from each population were initially grown in a common growth chamber environment (details in Lacey et al., 2010). After 10 weeks, each plant was divided into two genetically identical clones and maintained in the common environment to recover. Then, each clone was randomly assigned to either a “cool” growth chamber set to 15°C day/10°C night or a “warm” chamber set to 27°C day/20°C night, and flowering was induced by increasing the day‐length. These temperatures are within the natural range experienced by populations during reproduction (Lacey et al., 2010). For each clone, reflectance was estimated as the percentage of light reflected from flower buds (i.e., spikes just before stigma emergence on the lowest flowers) at 850 nm (in NIR region) using a spectrophotometer with an integrated sphere (details in Lacey & Herr, 2005). An individual's (i.e., genotype's) thermal plasticity in floral reflectance was calculated by subtracting percent reflectance of spikes produced by a clone grown at cool temperature from the percent reflectance of spikes produced by a clone of the same genotype grown at warm temperature (for complete methodology, see Lacey & Herr, 2005; Lacey et al., 2010). One‐sided Pearson correlations showed that the geographic patterns of thermal plasticity in our sample populations were consistent with the overall geographic pattern observed in the earlier, larger study (Lacey et al., 2010). Mean plasticity increased significantly with latitude (*r* = 0.528, one‐sided *p* = 0.026) and marginally with altitude (*r* = 0.418, one‐sided *p* = 0.068) of the source population (R Development Core Team, 2013). We used a one‐sided test to test the directional hypothesis of a positive correlation.

### 2.3 Phenotypic differentiation

Phenotypic differentiation (*P*_ST_) in thermal plasticity was calculated as a conservative proxy for quantitative genetic differentiation (*Q*_ST_) associated with floral reflectance plasticity as

(1) $$P_{\text{ST}}=\frac{\sigma_{\text{PB}}^{2}}{\sigma_{\text{PB}}^{2}+\;2\left(h^{2}\sigma_{\text{PW}}^{2}\right)\;}\;,$$

where $\sigma_{\text{PB}}^{2}$ denotes between‐population phenotypic variance, $\sigma_{\text{PW}}^{2}$ within‐population phenotypic variance, and *h* ^2^ the heritability (Leinonen, Cano, Mäkinen, & Merilä, 2006; Merilä & Crnokrak, 2001). We calculated *P*_ST _using the null assumption that *h^2^*=1, that is, all of the observed phenotypic variation is genetic, for two reasons. First, the plants we used for phenotyping were second‐generation plants from each population that had been grown in a common environment, and the phenotypes were scored in a common environment. Thus, environmental effects from the previous two generations were controlled and should not have inflated the between‐population variance in our study. Second, we were unable to determine reliable estimates of heritability. By using the assumption *h^2^*=1, our measure of *P*_ST_ is a conservative proxy for *Q*_ST_. Phenotypic variance components were calculated for plasticity between each pair of populations using ANOVA tests in SPSS (Merilä & Crnokrak, 2001; SPSS, 2009). The 95% confidence intervals for phenotypic differentiation were determined from 200 bootstrapped *P*_ST_ values (R Development Core Team, 2013).

### 2.4 Neutral genetic markers

We extracted DNA from leaf tissue of 315 individuals (*n* = 10–33 individuals/population) using a modified CTAB method (Doyle & Dickson, 1987) and prepared amplified fragment length polymorphism (AFLP) reaction templates following Vos et al. (1995) using 500 ng of DNA digested with *Eco*RI and *Mse*I. Thereafter, we completed ligation with *Eco*RI (E) and *Mse*I (M) primers and selective preamplification using standard AFLP *Eco*RI (E) and *Mse*I (M) primers containing selective nucleotides E + AC and M + CC (Remington, Whetten, Liu, & O'malley, 1999; Vos et al., 1995). Selective amplification was performed using combinations of the following E primer with three selective nucleotides and M primers with four selective nucleotides (E + 3/M + 4), *Eco*RI primer E + ACC labeled with one of the fluorescent dyes FAM or TAMRA, in combination with each of the selective *Mse*I primers M + CCAA, M + CCAT, M + CCAC, M + CCAG, M + CCTA, M + CCTT, M + CCTC, M + CCTG. As such, AFLP fragments from each individual were produced using each primer‐pair combination and either the FAM or TAMRA dye. We pooled DNA samples of fragments from the same individuals but with different dye and primer combinations into the same well for desalting and fragment detection (e.g., selective amplification products from E + ACC + FAM/M + CCTA and E + ACC + TAMRA/M + CCTC were pooled for individuals 1–48). We quantified AFLP reaction products with MegaBACE ET550‐R size standards on a MegaBACE^TM^ Fragment Profiler and scored them in GeneMarker (Softgenetics).

We used individuals repeated within each primer‐pair combination to establish consensus AFLP scoring panels, and all loci were repeated with at least 5 individuals. All of the individuals that were genotyped with multiple dyes were used to create scoring panels. In cases where one of the samples of repeated individuals was too poor to score, and the other sample was used for scoring. In cases of disagreement, the sample with the clearest standards in that region was used. If both samples were of equal quality, disagreements were treated as missing data. In total, we scored 313 unique AFLP loci in each of 315 individuals.

Within AFLP scoring panels, we determined scoring error for each dye, and between dyes (Supporting Information Table S1). We calculated scoring error in AFLP markers as percent of markers that disagreed (percent disagreement) between multiple runs of an individual within and between FAM and TAMRA dyes. We calculated error within each primer‐pair combination as the number of markers in disagreement divided by the total number of markers able to be scored within repeated individuals. To calculate overall error, we summed the average error within all primer pairs, weighted by the number of individuals used, and divided by the total number of individuals used. The percent of AFLP markers that disagreed between multiple runs of the same individual were 5.06 ± 1.79% and 5.51 ± 2.29% for FAM and TAMRA dyes and 8.86 ± 1.67% between dyes (Supporting Information Table S1).

Once AFLP scoring panels were established, they were used to score each individual. In the final AFLP data set, each individual was included once for each primer pair (individuals were not repeated). Using the criteria described above, we developed a consensus score for individuals for which we had data from multiple runs.

### 2.5 Environmental variables

We considered the following four environmental characteristics of the reproductive season for our analyses. (a) The "cool proportion" of the season was calculated as the proportion of the flowering season spent at temperatures below 15°C, based on monthly means. The physiological rationale for this value is described in Lacey et al. (2010). (b) The reproductive “duration,” the phenological window within which a plant reproduces, was calculated as the length of the flowering season in months. Lacey et al. (2010) estimated reproductive duration using flowering phenology data obtained from published guides to local flora (Davies & Gibbons, 1993; Godet, 2002), personal observation (E.P. Lacey) and personal communication with biologists who collected parental seeds for the study. (c) The “magnitude” of thermal variation was calculated as the absolute value of mean monthly maximum temperature minus mean monthly minimum temperature during the flowering season. (d) Precipitation was calculated as the mean monthly total precipitation during the flowering season. Climatic variables were extracted from the Climatic Research Unit Global Climate data set as 30‐year averages (1961–1990) of monthly means (www.ipcc-data.org). Monthly means were used because daily data were not available. The period 1961–1990 was selected to reflect the environmental conditions under which the populations we sampled had evolved. Variables were estimated by interpolation of the nearest neighboring weather stations to each population (complete methodology in Lacey et al., 2010). We did not use latitude or altitude as predictor variables because they do not directly estimate environmental conditions.

To simultaneously estimate the combined effect of multiple variables, we created three composite reproductive environment variables using principal components analyses (PCA) (*prcomp *function, R Development Core Team, 2013). The first, Thermal_PC, included the cool proportion and duration. The second, Magnitude_PC, included the magnitude and duration. The third, Mag_Therm_PC, included the magnitude, cool proportion, and duration. These composite variables were used to assess the combined effect of multiple factors in MRM and linear regression analyses. For each PCA, the first principal component axis explained the majority (>80%) of the variance, and this axis alone was used in subsequent analyses (Table 2). Factor loadings indicated that: more positive Thermal_PC values represented longer reproductive seasons with a smaller cool proportion of time; more positive Magnitude_PC values represented longer reproductive seasons with more thermal variation; and more positive Mag_Therm_PC values represented longer reproductive seasons with a greater magnitude of thermal variation and a smaller proportion of time at cool temperatures (Table 2).

**Table 2:** Principal components analyses used to combine multiple aspects of the reproductive season into composite variables

	% Explained	Eigenvalue	Factor loadings		
			DegMoB15°C	Magnitude	Duration
Thermal_PC					
PC1	83.2	1.66	−0.707	—	0.707
PC2	16.8	1.34	0.707	—	0.707
Magnitude_PC					
PC1	92.7	1.85	—	0.707	0.707
PC2	7.3	0.15	—	−0.707	0.707
Mag_Therm_PC					
PC1	81.5	2.45	−0.539	0.593	0.598
PC2	13.6	0.41	−0.841	−0.409	−0.353
PC3	4.9	−0.03	−0.035	0.693	−0.720

Note

Thermal_PC combines proportion of the reproductive season under 15°C (DegMoB15°C) and season duration, Magnitude_PC combines thermal magnitude and season duration, and Mag_Therm_PC combines proportion of the reproductive season under 15°C, thermal magnitude, and season duration. Only the primary axis was used in subsequent analyses.

Finally, we calculated absolute pairwise differences between populations for each environmental variable and for each composite variable.

### 2.6 Analyses

#### 2.6.1 Neutral genetic population structure

Scored AFLP markers were used to estimate genetic diversity within populations and differentiation between populations and to conduct population structure analyses. We estimated neutral genetic diversity as population mean heterozygosity for each population in Hickory v1.1 with 10^5^ iterations following a burn‐in of 5,000 (Holsinger, Lewis, & Dey, 2002). Comparative phylogeographic studies have found evidence of postglacial migration from southern European refugia following the Pleistocene glaciation in other species (Schönswetter, Stehlik, Holderegger, & Tribsch, 2005; Taberlet, Fumagalli, Wust‐Saucy, & Cosson, 1998). To determine whether we could identify postglacial migration routes in *P. lanceolata*, potentially providing information on the origins of neutral patterns of genetic differentiation, we mapped diversity at population locations and looked for emerging patterns (Supporting Information Figure S1). Two‐sided Pearson correlations between heterozygosity with latitude and altitude were calculated in R (Goslee & Urban, 2007; R Development Core Team, 2013).

We calculated neutral genetic differentiation and 95% confidence intervals between all population pairs using two statistics; *F*_ST_ estimated with the full model as θ^II^ in Hickory v1.1 with 10^5^ iterations following a burn‐in of 5,000, and Jost's *D* calculated in SPADE using 300 bootstraps (Chao & Schen, 2010; Holsinger et al., 2002; Jost, 2008). *Q*_ST_ or *P*_ST_ and *F*_ST_ statistics are equivalent measures of population phenotypic and genetic differentiation and thus are derived from the same evolutionary history and respond similarly to the evolutionary processes that give rise to them (i.e., realized migration and genetic drift). Jost's *D* on the other hand is specific to the loci being measured and is more strongly affected by mutation than migration. Thus, Jost's *D* is not legitimately equivalent to *Q*_ST_ or *P*_ST_ (Whitlock, 2011). However, we included Jost's *D* because it is better suited for describing the allelic differentiation among populations (Meirmans & Hedrick, 2011).

#### 2.6.2 Admixture

To explore the genetic groups within samples and infer geographic patterns of admixture across the landscape, nonhierarchical Bayesian clustering was performed in STRUCTURE 2.3.4 (Pritchard, Stephens, & Donnelly, 2000). The correlated allele frequencies model with admixture was used to test values of *K*. Five replicates for each K from 2–10 were run with a burn‐in of 10^5^, followed by 10^6^ replicates, with convergence monitored for each run. We combined and interpreted all runs with Structure Harvester (Earl, 2012), using the methods of Evanno, Regnaut, and Goudet (2005) and Pritchard et al. (2000). We used CLUMPP to average admixture proportions over runs (Jakobsson & Rosenberg, 2007) and visualized averaged runs using Distruct (Rosenberg, 2004). To best resolve ancestral relatedness among populations, we visually examined average admixture plots from low to high values of K groups, with regard to the geographic location of populations.

Then, we grouped populations into geographic regions based upon both their physical locations and the groupings provided by STRUCTURE, and we calculated genetic differentiation between these regions in Hickory with 25,000 iterations following a burn‐in of 5,000 (Holsinger et al., 2002). We looked for regions separated by higher genetic differentiation that may represent ancestral populations, and regions separated by lower differentiation representing historical postglacial migration routes (Fischer, 1960; Hewitt, 1999; Schmitt, 2007).

### 2.7 Hypothesis testing

Phenotypic, genetic, and environmental differentiation values were standardized to zero mean and unit variance with the *decostand *function (R Development Core Team, 2013). Standardized values were used for hypothesis testing in subsequent analyses.

### 2.8 Isolation by distance

To determine whether isolation by distance could explain geographic patterns of differentiation in neutral genetic markers, we conducted Mantel tests (10^6^ permutations) between neutral genetic differentiation (*F*_ST_ and Jost's *D*) and geographic distance between populations in the *ecodist* package (Goslee & Urban, 2007; R Development Core Team, 2013). The null hypothesis of the Mantel test is that differentiation matrices are uncorrelated. The simple Mantel test is suitable for testing the absence of IBD from population genetic data (Guillot & Rousset, 2013) and for addressing questions that concern dissimilarity matrices (Legendre, Fortin, & Borcard, 2015). However, Mantel test results should be interpreted cautiously because simulations have shown them to reject the null hypothesis of independence too often, producing a higher number of false positives than it should when comparing two nonspatial matrices that might both be spatially autocorrelated (Guillot & Rousset, 2013).

To determine whether isolation by distance could explain a significant proportion of the variance in thermal plasticity, we performed multiple regressions on distance matrices (MRM) of phenotypic differentiation (*P*_ST_) on genetic differentiation (*F*_ST_ and Jost's *D*) and geographic distance with 10^6^ permutations in *ecodist* (Goslee & Urban, 2007; R Development Core Team, 2013). The MRM models and regression coefficients were tested by permuting the dependent distance matrix (i.e., phenotypic differentiation of thermal plasticity) while holding the explanatory matrices constant (Lichstein, 2007).

### 2.9 Natural selection

To test for evidence of natural selection, we compared phenotypic and neutral genetic differentiation. We examined the relationship between phenotypic (*P*_ST_) and neutral genetic (*F*_ST_) differentiation by examining whether 95% confidence intervals for *P*_ST_ and for *F*_ST_ among population pairs overlapped the value where *F*_ST_ = *P*_ST_. When 95% confidence intervals failed to overlap the value where *F*_ST_ = *P*_ST_, we concluded *F*_ST_ and *P*_ST_ differed. We could not utilize more advanced methods developed for predicting the distribution of neutral *P*_ST_ values based on *F*_ST_ values (e.g., Gilbert & Whitlock, 2015; Whitlock & Guillaume, 2009), because available packages for calculating *F*‐statistics from dominant AFLP markers do not provide the required per‐locus components of variance, that is, the coefficients “*a*”, “*b*”, and “*c*” from Weir and Cockerham (1984).

To determine whether environmental properties of the reproductive season explained a significant proportion of the variation in phenotypic differentiation in thermal plasticity, we used Mantel tests and MRM. The independence of geographic distance and environmental properties of the reproductive season were determined using Mantel tests with 10^6^ permutations in the *ecodist* package (Goslee & Urban, 2007; R Development Core Team, 2013). We were unable to address the environment–genetic relationship using alternative analytical methods (e.g., spatial regression, multivariate ordination) because methods to reduce our genetic differentiation statistics into a single linear genotypic variable are not available for these purposes. Putting genetic differentiation on a linear scale via PCA would only capture the biggest parts of the genetic differentiation, not all of it, making the comparisons with phenotypic differentiation and most of the other analyses invalid. Therefore, we performed MRM analyses of phenotypic differentiation (*P*_ST_) on genetic differentiation (*F*_ST_ and Jost's *D*) and each environmental variable independently with 10^6^ permutations in *ecodist *(Goslee & Urban, 2007; R Development Core Team, 2013). All models were conducted with MRM to allow for ease of comparison among models.

To examine the patterns of between‐population differences in *P*_ST_, *F*_ST_, geographic distance, and each environmental variable, we used linear regression analyses. Linear regression allowed us to determine whether (a) phenotypic and genetic differentiation increased with increasing geographic distance and along environmental clines, and (b) slopes and y‐intercepts of phenotypic and genetic differentiation differed along these axes. In cases where the slope of phenotypic differentiation was significantly greater than that of neutral genetic differentiation, there was no need to test for equality of *y*‐intercepts. Data in each linear regression were tested for linearity using a runs test. We regressed phenotypic differentiation (*P*_ST_) and genetic differentiation (*F*_ST_ and Jost's *D*) against axes of geographic distance between populations, and pairwise‐population differences in environmental properties of the reproductive season (i.e., cool proportion, duration, magnitude, and precipitation) using GraphPad Prism version 6.07 for Windows, GraphPad Software, La Jolla California USA, www.graphpad.com.

The local adaptation hypothesis predicts that phenotypic differentiation should diverge more rapidly than neutral genetic differentiation along selective gradients. Therefore, under natural selection, *P*_ST_ should (a) increase more sharply than *F*_ST_ and Jost's *D* as environments increasingly differ between populations and/or (b) have a higher y‐intercept value (Leinonen et al., 2006; Orsini et al., 2013). Although the magnitude and frequency hypotheses are not mutually exclusive, each predicts that different characteristics of the reproductive season for *P. lanceolata *drive the evolution of thermal plasticity. The Magnitude Hypothesis predicts that the highest thermal plasticity of floral reflectance is associated with the most thermally variable reproductive seasons. The Frequency Hypothesis predicts that thermal plasticity of floral reflectance is greatest where reproductive seasons are coolest and shortest.

To control for nonindependence of population pairs, we used a jackknifing procedure to further test whether the slope of phenotypic differentiation was significantly greater than the slope for genetic differentiation. This was accomplished by conducting a linear regression to estimate the slopes and *p*‐values 91 additional times. Before each regression, one of the 91 pairwise comparisons was eliminated from the analysis. We eliminated a different comparison each time. The means and standard deviations of the jackknifing procedure are reported.

Finally, we controlled for the false discovery rate (FDR) at *α* = 0.05 among MRM models using the two‐staged sharpened method because the graphically sharpened procedure was unable to estimate adjusted *p*‐values (i.e., *q‐values*; Benjamini, Krieger, & Yekutieli, 2006). For all other analyses, we controlled for FDR, adjusted *p*‐values, and determined their significance using the graphically sharpened method for multiple comparisons (Benjamini & Hochberg, 2000). Adjusted *p*‐values are reported.

## 3 RESULTS

### 3.1 Neutral genetic population structure is consistent with gene flow among populations

**Figure 2:**
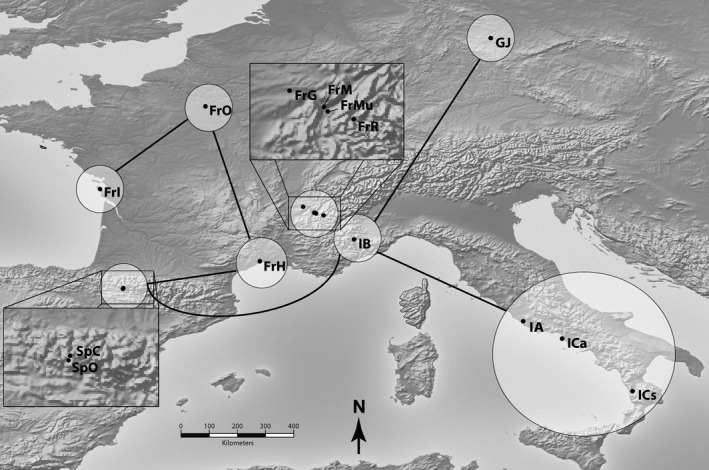
Map of European *Plantago lanceolata* populations showing genetic similarity and differentiation calculated in Hickory. Circles represent geographic regions between‐which *F*_ST _(estimated as *θ* ^II^ in Hickory) values were calculated. Lines connect genetically similar regions with *F*_ST_ < 0.09, consistent with ancestral or contemporary gene flow among populations. Values of *F*_ST_ < 0.09 delineate populations into spatially separated regions. Population symbols identified in Table 1. Pop‐out boxes are zoomed 4×

Spatial patterns of admixture and heterozygosity are consistent with ancestral or contemporary gene flow having occurred among European populations of *P. lanceolata*. At the AFLP markers we sampled, mean population heterozygosity did not vary with latitude (*t* = −0.583, *p* = 0.571) or altitude (*t* = −0.222, *p* = 0.828). The northern Italian population IB, which displayed the greatest overall heterozygosity, was genetically more similar to populations in Germany, southern Italy, and Spain than to other populations (*F*_ST_ ≤ 0.021, Figure 2, Supporting Information Table S2). Relatively low genetic differentiation was found between several regions: Spain and southern France, southern and northern France, and northern and western France (0.05 ≤ *F*_ST_ ≤ 0.10, Figure 2, Supporting Information Table S2).

**Figure 3:**
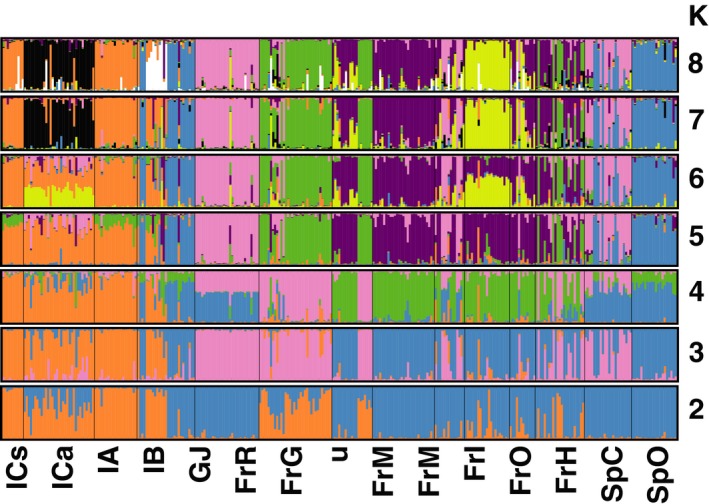
STRUCTURE admixture plots of 14 *Plantago lanceolata* populations from southern Europe calculated with 313 AFLP markers from 315 individuals. CLUMPP was used to average admixture proportions over runs (Jakobsson & Rosenberg, 2007) and Distruct (Rosenberg, 2004) for visualization

STRUCTURE indicated that the best arrangement for AFLP data from the 14 European populations was in *K* = 7 or 8 groups. The highest delta *K* value was observed at *K* = 7, while *K* = 8 showed the highest log probability and low run‐to‐run variability (Supporting Information Figures S2 and S3). As values of *K* increased from 2 to 8, evidence of admixture among populations also increased (i.e., new groups were spread among multiple populations). Despite the admixture, southern Italian populations (IA, ICa, and ICs) consistently remained different from other populations (Figure 3). This pattern was noticeable at *K* = 2 and *K* = 8 (Figure 3).

### 3.2 Isolation by distance

Analyses conducted with neutral genetic differentiation estimated as *F*_ST_ and Jost's *D* produced similar conclusions. Geographic distance between populations always had a strong influence on phenotypic differentiation. Also, *F*_ST_ made a difference only to the extent that it was a partial proxy for geographic distance. Neutral genetic differentiation among populations was strongly associated with geographic distance (*F*_ST_: *r* = 0.602, *p* < 0.001; Jost's *D*: *r* = 0.640, *p* < 0.001). In MRM analyses that excluded geographic distance, neutral genetic differentiation (*F*_ST_ and Jost's *D*) was always a statistically or marginally significant predictor of the variation in *P*_ST_ (Tables 3B,F,J,N,R and 4C,G,K, Jost's *D* results in Supporting Information Tables S3 and S4). However, when geographic distance was included in the model, *F*_ST_ and Jost's *D* became nonsignificant (Tables 3C,G,K,O,S and 4D,H,L, Jost's *D* results in Supporting Information Tables S3 and S4). Removing *F*_ST_ from models with both geographic distance and *F*_ST_ had little effect on the variation explained (e.g., Table 3C vs. 3A), whereas removing geographic distance from these models substantially reduced the variation explained (e.g., Table 3C vs. 3B). Thus, on its own, *F*_ST_ was a poor predictor of phenotypic differentiation.

**Table 3:** Results of multiple regression of distance matrices (MRM) tests of the phenotypic differentiation (*P*_ST_) in temperature‐sensitive floral reflectance plasticity matrix on matrices of geographic distance, genetic differentiation (*F*_ST_), and/or environmental differences between *Plantago lanceolata* populations

MRM Model	*r* ^2^	Intercept	Distance	*F*_ST_	Duration	DegMoB15°C	Magnitude	Precipitation
A. *P*_ST_ ~ Distance	0.17 **(0.006)**	2.35E−11 (0.962)	4.32E−01 **(0.006)**	**—**	**—**	**—**	**—**	**—**
B. *P*_ST_ ~ *F*_ST_	0.11 **(0.006)**	3.30E−11 **(0.019)**	**—**	3.27E−01 **(0.019)**	**—**	**—**	**—**	**—**
C. *P*_ST_ ~ *F*_ST_ + Distance	0.18 **(0.006)**	2.94E−11 *(0.072)*	3.28E−01 **(0.032)**	1.29E−01 (0.403)	**—**	**—**	**—**	**—**
D. *P*_ST_ ~ Duration	0.02 **(0.048)**	3.74E−11 (0.115)	**—**	**—**	1.34E−01 (0.188)	**—**	**—**	**—**
E. *P*_ST_ ~ Distance + Duration	0.18 **(0.006)**	3.24E−11 (0.240)	4.02E−01 **(0.006)**	**—**	1.18E−01 (0.215)	**—**	**—**	**—**
F. *P*_ST_ ~ *F*_ST_ + Duration	0.11 **(0.011)**	3.58E−11 (0.122)	**—**	3.15E−01 **(0.026)**	8.51E−02 (0.408)	**—**	**—**	**—**
G. *P*_ST_ ~ *F*_ST_ + Distance + Duration	0.19 **(0.006)**	3.27E−11 (0.240)	3.37E−01 **(0.028)**	1.09E−01 (0.490)	1.03E−01 (0.288)	**—**	**—**	**—**
H. *P*_ST_ ~ DegMoB15°C	0.06 **(0.010)**	2.76E−11 (0.967)	**—**	**—**	**—**	2.43E−01 **(0.033)**	**—**	**—**
I. *P*_ST_ ~ Distance + DegMoB15°C	0.20 **(0.006)**	2.44E−11 (0.983)	3.84E−01 **(0.007)**	**—**	**—**	1.98E−01 **(0.048)**	**—**	**—**
J. *P*_ST_ ~ *F*_ST_ + DegMoB15°C	0.14 **(0.007)**	2.89E−11 (0.836)	**—**	2.90E−01 **(0.036)**	**—**	1.83E−01 *(0.069)*	**—**	**—**
K. *P*_ST_ ~ *F*_ST_ + Distance + DegMoB15°C	0.21 **(0.006)**	2.53E−11 (0.961)	3.30E−01 **(0.029)**	9.10E−02 (0.557)	**—**	1.86E−01 *(0.060)*	**—**	**—**
L. *P*_ST_ ~ Magnitude	0.02 *(0.064)*	3.45E−11 (0.139)	**—**	**—**	**—**	**—**	1.35E−01 (0.253)	**—**
M. *P*_ST_ ~ Distance + Magnitude	0.19 **(0.006)**	3.02E−11 (0.412)	4.19E−01 **(0.005)**	**—**	**—**	**—**	1.66E−01 (0.109)	**—**
N. *P*_ST_ ~ *F*_ST_ + Magnitude	0.13 **(0.010)**	3.44E−11 (0.060)	**—**	3.28E−01 **(0.018)**	**—**	**—**	1.35E−01 (0.227)	**—**
O. *P*_ST_ ~ *F*_ST_ + Distance + Magnitude	0.20 **(0.006)**	3.09E−11 (0.332)	3.47E−01 **(0.022)**	1.19E−01 (0.442)	**—**	**—**	1.61E−01 (0.120)	**—**
P. *P*_ST_ ~ Precipitation	0.01 (0.120)	3.43E−11 (0.258)	**—**	**—**	**—**	**—**	**—**	−1.20E−01 (0.488)
Q. *P*_ST_ ~ Distance + Precipitation	0.17 **(0.006)**	2.86E−11 (0.838)	4.05E−01 **(0.007)**	**—**	**—**	**—**	**—**	−5.90E−03 (0.972)
R. *P*_ST_ ~ *F*_ST_ + Precipitation	0.11 **(0.014)**	3.37E−11 (0.133)	**—**	3.17E−01 **(0.025)**	**—**	**—**	**—**	−6.62E−02 (0.704)
S. *P*_ST_ ~ *F*_ST_ + Distance + Precipitation	0.18 **(0.007)**	2.94E−11 (0.701)	3.27E−01 **(0.035)**	1.30E−01 (0.408)	**—**	**—**	**—**	−5.80E−03 (0.973)

Note

Environmental variables examined were reproductive season duration (Duration), the proportion of the reproductive season having temperatures less than 15°C (DegMoB15°C), the magnitude of thermal variation during the reproductive season (Magnitude) and total reproductive season precipitation (Precipitation). MRM model *r* ^2^ and regression coefficients obtained from permutation tests are reported. FDR‐adjusted *p*‐values are listed parenthetically: bold = *p* < 0.05, italic = 0.05 < *p* < 0.10. See Section 2 for more information about MRM tests.

**Table 4:** Results of multiple regression of distance matrices (MRM) tests of the phenotypic differentiation (*P*_ST_) in temperature‐sensitive floral reflectance plasticity matrix on matrices of geographic distance, genetic differentiation (*F*_ST_), and/or environmental differences between *Plantago lanceolata* populations

MRM model	*r* ^2^	Intercept	Distance	*F*_ST_	Thermal_PC	Magnitude_PC	Mag_Therm_PC
A. *P*_ST_ ~ Thermal_PC	0.08** (0.007)**	2.99E−11 (0.984)	**—**	**—**	0.28** (0.022)**	**—**	**—**
B. *P*_ST_ ~ Distance + Thermal_PC	0.21** (0.006)**	2.65E−11 (0.994)	0.371** (0.008)**	**—**	0.219** (0.044)**	**—**	**—**
C. *P*_ST_ ~ *F*_ST_ + Thermal_PC	0.15** (0.006)**	3.07E−11 (0.568)	**—**	0.269* (0.054)*	0.203* (0.074)*	**—**	**—**
D. *P*_ST_ ~ *F*_ST_ + Distance + Thermal_PC	0.22** (0.006)**	2.71E−11 (0.949)	0.331** (0.029)**	0.0693 (0.659)	0.206 (*0.062*)	**—**	**—**
E. *P*_ST_ ~ Magnitude_PC	0.02** (0.048)**	4.28E−11 (0.114)	**—**	**—**	**—**	0.149 (0.185)	**—**
F. *P*_ST_ ~ Distance + Magnitude_PC	0.19** (0.006)**	3.84E−11 (0.137)	0.407** (0.005)**	**—**	**—**	0.150 (0.143)	**—**
G. *P*_ST_ ~ *F*_ST_ + Magnitude_PC	0.12** (0.011)**	4.14E−11 (0.109)	**—**	0.319** (0.022)**	**—**	0.128 (0.241)	**—**
H. *P*_ST_ ~ *F*_ST_ + Distance + Magnitude_PC	0.20** (0.006)**	3.86E−11 (0.137)	0.338** (0.027)**	0.115 (0.460)	**—**	0.143 (0.168)	**—**
I. *P*_ST_ ~ Mag_Therm_PC	0.07** (0.01)**	2.73E−11 (0.965)	**—**	**—**	**—**	**—**	0.259** (0.035)**
J. *P*_ST_ ~ Distance + Mag_Therm_PC	0.21** (0.006)**	2.4E−11 (0.987)	38.3** (0.006)**	**—**	**—**	**—**	0.217* (0.053)*
K. *P*_ST_ ~ *F*_ST_ + Mag_Therm_PC	0.15** (0.006)**	2.85E−11 (0.825)	**—**	0.288** (0.037)**	**—**	**—**	0.202* (0.081)*
L. *P*_ST_ ~ Distance + *F*_ST_ + Mag_Therm_PC	0.22** (0.006)**	2.48E−11 (0.968)	0.331** (0.028)**	0.088 (0.569)	**—**	**—**	0.205* (0.069)*

Note

Environmental variables shown here are principal component axes that combined multiple variables. Thermal_PC combined duration and the proportion of the reproductive season having temperatures less than 15°C. Magnitude_PC combined duration and the magnitude of thermal variation during the reproductive season. Mag_Therm_PC combined duration, proportion of the reproductive season having temperatures less than 15°C, and seasonal magnitude of thermal variation. MRM model *r* ^2^ and regression coefficients obtained from permutation tests are reported. FDR‐adjusted *p*‐values are listed parenthetically: bold = *p* < 0.05, italic = 0.05 < *p* < 0.10. See Section 2 for more information about MRM tests.

Our data were able to distinguish the influence of geographic distance on phenotypic differentiation separately from contributions of potentially selective environmental variables because pairwise differentiation in environmental parameters and geographic distance was uncorrelated (Supporting Information Table S5). As a result, the significance of environmental variables as predictors of variation in *P*_ST_ in MRM models was influenced only modestly by including geographic distance in models (e.g., Table 3H vs. 3I).

#### 3.2.1 *P*_ST_ analyses

**Figure 4:**
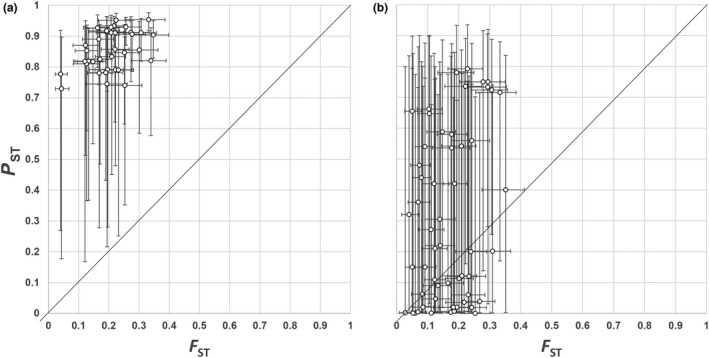
Scatter plot of population pairwise comparisons between phenotypic differentiation (*P*_ST_) of temperature‐sensitive floral reflectance plasticity and neutral genetic differentiation (*F*_ST_) ± 95% CI between 14 *Plantago lanceolata *populations. (a) 36 of 91 comparisons where 95% CI for *P*_ST_ and for *F*_ST_ did not overlap the *P*_ST_ = *F*_ST_ line, indicating *P*_ST_ > *F*_ST_. (b) 55 of 91 comparisons showed 95% CI for *P*_ST_ or for *F*_ST_ overlapped the *P*_ST_ = *F*_ST_ line, indicating statistical support for a difference was lacking. The diagonal line indicates *P*_ST_ = *F*_ST_

For 36 of the 91 (39.6%) population pairwise comparisons, we found that *P*_ST_ was greater than neutral genetic differentiation (*F*_ST_) and the 95% confidence intervals did not include values where *P*_ST_ = *F*_ST_ (Figure 4). We did not find statistical support for a difference among the remaining comparisons, and no comparison suggested that *F*_ST_ > *P*_ST_.

MRM models showed that a noteworthy fraction of the variation in *P*_ST_ could be explained by the cool proportion of the season and Thermal_PC (the composite variable for cool proportion and duration), independent of the contributions of geographic distance and *F*_ST_ (Tables 3H–K and 4A–D). In models including cool proportion and geographic distance, cool proportion significantly or marginally contributed to the variation in *P*_ST_ (Table 3I,K). In the model including Thermal_PC and distance, Thermal_PC significantly contributed to the variation in *P*_ST_ (Table 4B), and the contribution was marginal when *F*_ST_ was added to this model (Table 4D). Overall, the models that included Thermal_PC had the highest predictive power.

Substituting Mag_Therm_PC for Thermal_PC (i.e., adding magnitude to the composite environmental variable) failed to improve the model's explanatory power when compared to the model with Thermal_PC alone (Table 4A–D vs. 4I–L). Associations with duration, magnitude, and precipitation alone were not significant (Table 3D–G, L–O, P–S).

**Figure 5:**
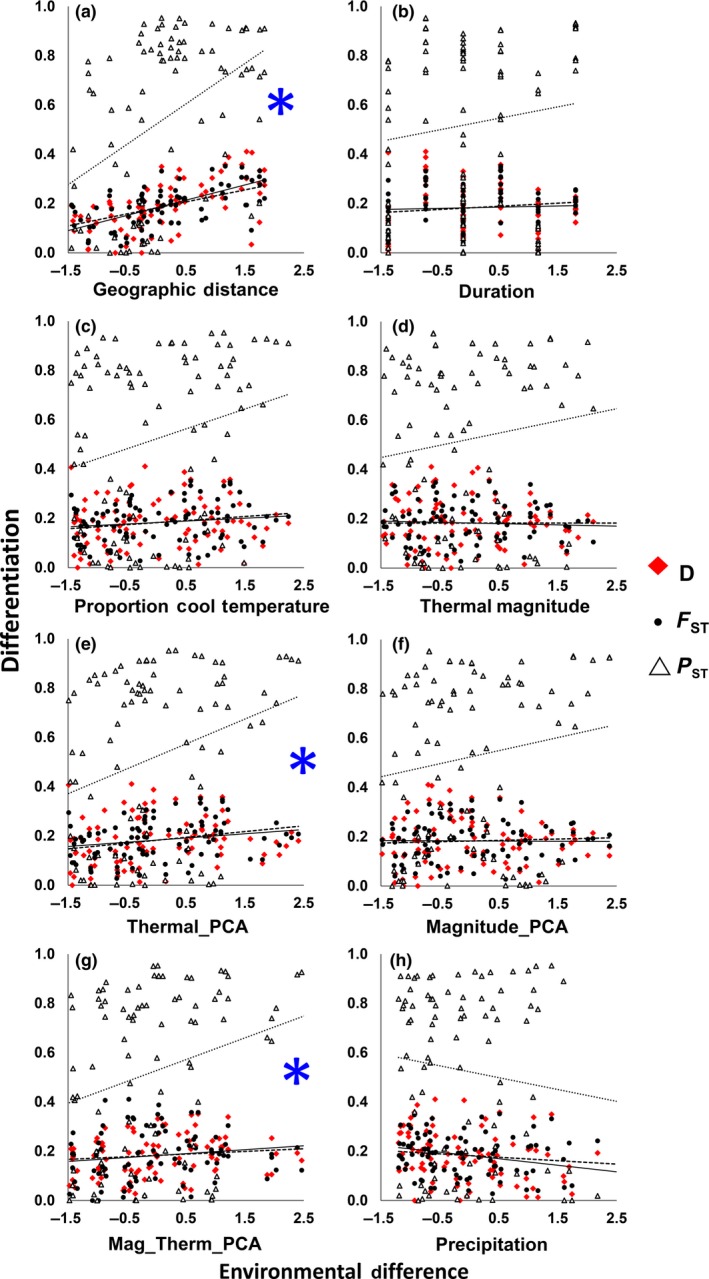
Linear regressions of phenotypic differentiation (*P*_ST_, triangle, dotted line) of temperature‐sensitive floral reflectance plasticity and neutral genetic differentiation (*F*_ST_, circle, dashed line; Jost's *D*, diamond, solid line) on an axis (*x*) of increasing standardized environmental difference between 14 *Plantago lanceolata* populations. Along the *x*‐axis (a) geographic distance, or environmental properties of the reproductive season diverge between populations from left to right. Environmental variables are (b) season duration, (c) proportion of the season below 15°C, (d) season thermal magnitude, (h) total season precipitation, and principal components axes (e) Thermal_PC, (f) Magnitude_PC, (g) Mag_Therm_PC. * indicates where the slope of *P*_ST_ is significantly greater than the slopes for *F*_ST_ and *D*

The linear regression analyses showed that the slopes of P_ST_ differed substantially from *F*_ST_ and Jost's *D* when the statistics were regressed on geographic distance between populations and on population differences for two environmental variables characterizing the reproductive season (Table 5). *P*_ST_, *F*_ST_, and Jost's *D* increased with increasing geographic distance between populations, but *P*_ST_ increased at a significantly greater rate than did *F*_ST_ and Jost's *D* (Figure 5a, Table 5). In contrast, only *P*_ST_ increased significantly with increasing population differences in the proportion of cool temperatures during reproductive season (Figure 5c, Table 5). With respect to population differences in duration of the reproductive season, and also proportion of cool temperatures, the slope of *P*_ST_ did not significantly differ from *F*_ST_ or Jost's *D*, but the y‐intercept was significantly greater for *P*_ST_ than for *F*_ST_ and Jost's *D* (Figure 5b,c, Table 5). As population differences in Thermal_PC (cool proportion and duration variables combined) increased, so also did *P*_ST_ and *F*_ST_ (Figure 5e, Table 5). The slopes for both *P*_ST_ and *F*_ST_ were significantly positive, but the slope for *P*_ST_ was significantly steeper than for *F*_ST_ (Figure 5e, Table 5). Likewise, the slope for *P*_ST_ was significantly steeper than for *F*_ST_ along the Mag_Therm_PC axis (Figure 5g, Table 5).

**Table 5:** Linear regressions of phenotypic differentiation (*P*_ST_) in temperature‐sensitive floral reflectance plasticity and neutral genetic differentiation (*F*_ST_ and Jost's *D*) on either increasing geographic distance, among‐population differences in an environmental variable, or differences in a principal component variable that combines multiple environmental variables

Independent variable		*P*_ST_	*F*_ST_	Slope *P*_ST_ versus *F*_ST_	Intercept *P*_ST_ versus *F*_ST_	Jost's *D*	Slope *P*_ST_ versus *D*	Intercept *P*_ST_ versus *D*
Geographic distance	Slope	0.16	0.05	**—**	**—**	0.06	**—**	**—**
	*F*	25.69 **(<0.001)**	49.67 **(<0.001)**	2.13 **(<0.001)**	**—**	56.58 **(0.004)**	9.39 **(0.003)**	**—**
Reproductive season duration	Slope	0.05	0.01	**—**	**—**	0.00	**—**	**—**
	*F*	1.63 (0.133)	2.19 (0.116)	0.82 (0.366)	83.9 **(<0.001)**	0.21 (0.326)	1.21 (0.272)	81.3 **(<0.001)**
Proportion of reproductive season under 15°C	Slope	0.08	0.02	**—**	**—**	0.01	**—**	**—**
	*F*	5.09 **(0.037)**	3.87 (*0.064*)	3.04 (*0.083*)	88.95 **(<0.001)**	1.26 (0.161)	3.43 (*0.066*)	83.21 **(<0.001)**
Magnitude of thermal variation in reproductive season	Slope	0.05	0.00	**—**	**—**	−0.01	**—**	**—**
	*F*	1.86 (0.129)	0.00 (0.456)	1.75 (0.187)	83.55 **(<0.001)**	0.24 (0.326)	2.09 (0.150)	81.09 **(<0.001)**
Total precipitation in reproductive season	Slope	−0.05	−0.01	**—**	**—**	−0.03	**—**	**—**
	*F*	1.75 (0.130)	2.67 (0.097)	0.84 (0.361)	84.01 **(<0.001)**	6.92 (**0.018)**	0.33 (0.569)	82.25 **(<0.001)**
Thermal_PC	Slope	0.10	0.02	**—**	**—**	0.02	**—**	**—**
	*F*	8.27 **(0.013)**	8.03 (**0.013)**	4.62 **(0.033)**	**—**	2.75 (*0.097*)	5.25 **(0.023**)	**—**
Magnitude_PC	Slope	0.05	0.01	**—**	**—**	0	**—**	**—**
	*F*	2.13 (0.116)	0.40 (0.291)	1.62 (0.204)	83.87 **(<0.001)**	0.01 (0.440)	2.05 (0.154)	81.32 **(<0.001)**
Mag_Therm_PC	Slope	0.09	0.02	**—**	**—**	0.01	**—**	**—**
	*F*	6.38 **(0.020**)	3.53 (*0.070)*	4.05 **(0.046)**	**—**	1.07 (0.176)	4.51 **(0.035)**	**—**

Note

Thermal_PC combines duration and proportion of reproductive season under 15°C. Magnitude_PC combines duration and magnitude of thermal variation. Mag_Therm_PC combines duration, proportion of reproductive season under 15°C, and magnitude of thermal variation. *p*‐Values associated with differentiation statistics indicate whether the slope significantly deviates from zero. Comparisons test whether slopes or *y*‐intercepts differ between phenotypic and neutral genetic differentiation. In cases where slopes differed, we did not test for differences in *y*‐intercepts. FDR‐adjusted *p*‐values are reported parenthetically: bold = *p* < 0.05, italic = 0.05 < *p* <0.10.

Slopes for *P*_ST_ along axes of duration, thermal magnitude, precipitation and Magnitude_PC did not significantly differ from zero (Figure 5b,d,f,h, Table 5), and slopes for Jost's *D* were not significantly positive along any environmental principal components axis. In analyses where slopes did not differ between phenotypic and neutral genetic differentiation, the *y*‐intercepts of phenotypic differentiation were always higher than y‐intercepts of neutral genetic differentiation (Figure 5, Table 5).

Results of runs tests suggested phenotypic and genetic differentiation variables significantly deviated from linearity when regressed along the axis of reproductive season duration (Supporting Information Table S6). These deviations dissipated when season duration was incorporated into composite PC variables (Supporting Information Table S6). The jackknifing procedure that controlled for nonindependence of population pairs in linear regression analyses produced nearly identical results as those above, and conclusions were equivalent (Table 6).

**Table 6:** Jackknifing results of slopes and *p*‐values from linear regression analyses to control for nonindependence of population pairs in cases where the slope of phenotypic differentiation was significantly greater than the slope for genetic differentiation

		Linear regression values		Jackknifed values			
		Slope	*p*‐Values	Slope		*p*‐Values	
				Mean	St. Dev	Mean	St. Dev
A. Geographic distance	*P*_ST_	0.16	**<0.001**	0.16	0.003	2.67E−06	1.06E−06
	*F*_ST_	0.05	**<0.001**	0.05	7.10E−04	5.40E−10	2.90E−10
B. Proportion of season under 15°C	*P*_ST_	0.08	**0.027**	0.08	3.00E−03	0.028	0.006
	*F*_ST_	0.02	***0.052***	0.02	8.00E−04	0.054	0.012
C. Thermal_PC	*P*_ST_	0.10	**0.005**	0.10	0.003	0.006	0.002
	*F*_ST_	0.02	**0.006**	0.02	8.00E−04	0.006	0.002

Note

Our jackknife procedure estimated the slope and *p*‐values of each linear regression 91 additional times. Before each regression, one of the 91 pairwise comparisons was eliminated from the analysis. We eliminated a different comparison each time. The means, standard deviations, and *p*‐values of the jackknifing procedure are reported: bold = *p* < 0.05, italic = 0.05 < *p* < 0.10.

## 4 DISCUSSION

Our study is the first of which we are aware that has tested the local adaptation hypothesis against the “null” of neutral evolution for thermal plasticity in a trait. The results are consistent with natural selection having shaped large‐scale latitudinal and altitudinal patterns in thermal plasticity. Approximately 60% of the population pairwise *P*_ST_–*F*_ST_ comparisons did not provide statistical support for a difference between phenotypic differentiation and neutral genetic differentiation. Therefore, these differences presumably could be explained by genetic drift alone (McKay & Latta, 2002; Merilä & Crnokrak, 2001). However, phenotypic differentiation was greater than neutral differentiation among ~40% of the comparisons. Divergent selection better explains these differences (McKay & Latta, 2002; Merilä & Crnokrak, 2001). Moreover, multiple regression of distance matrices and linear regression analyses were consistent with phenotypic divergence in thermal plasticity occurring along clines in the reproductive environment.

Earlier attempts to test the local adaptation hypothesis against the null at large geographic scales have been limited in two ways. First, they have typically involved examining associations between phenotypic variation and composite environmental variables, for example, latitude (Chenoweth & Blows, 2008; Savolainen, Pyhäjärvi, & Knürr, 2007) and altitude (Luo, Widmer, & Karrenberg, 2015; Luquet et al., 2015). Thus, it has generally not been possible to identify the specific environmental drivers of selection (Hangartner et al., 2012; Muir et al., 2014; Orsini et al., 2013). Second, in most earlier *P*_ST_–*F*_ST_ comparisons, pairwise divergences in neutral genetic and environmental differentiation were strongly correlated with geographic distance (Hangartner et al., 2012; Muir et al., 2014; Nadeau et al., 2016; Orsini et al., 2013; Sexton, Hangartner, & Hoffmann, 2014). Thus, it was difficult to infer the independent contributions of neutral forces and environmental parameters in explaining population divergence. By examining associations with specific environmental parameters and by sampling a variety of populations over both latitudinal and altitudinal gradients, we were able to largely reduce both of the above limitations.

The results of MRM and linear regression analyses produced equivalent conclusions and were consistent with the prediction of the Frequency Hypothesis but not with that of the Magnitude Hypothesis (consistent with Lacey et al., 2010). Independent of the association between phenotypic differentiation and geographic distance, there was a marginally significant association between phenotypic differentiation and the cool proportion of the growing season and a statistically significant association between phenotypic differentiation and the composite variable Thermal_PC. The linear regression analyses showed that population phenotypic differentiation (*P*_ST_) increased more sharply than did neutral genetic differentiation (*F*_ST_ or Jost's *D*) with increasing differentiation in the relative proportion of reproductive time at cool temperatures. Additionally, combining the effect of the cool proportion and reproductive season duration variables into a single principal components axis, Thermal_PC, strengthened this effect, that is, increased the slope of *P*_ST_. In contrast, analyses showed no significant effect of the magnitude of thermal variation or precipitation.

Thermal plasticity was first proposed to be more adaptive at higher latitudes and altitudes because the magnitude of thermal variation is greater in these regions (Ghalambor et al., 2006; Janzen, 1967). The Magnitude Hypothesis (previously called climatic or temperature variability hypotheses) is consistent with mathematical models, and empirical data predicting that plasticity should be favored over nonplasticity as environmental variability increases (Gavrilets & Scheiner, 1993b; Kingsolver & Huey, 1998; Moran, 1992; Schlichting, 1986; Schlichting & Pigliucci, 1998; Via, 1993; Via & Lande, 1985). Alternatively, the Frequency Hypothesis is that thermal plasticity is more adaptive at higher latitudes and altitudes because in these regions, thermally variable growing seasons are shorter and experience a greater proportion of the growing season in cool, rather than warm temperatures relative to low latitude and altitude populations (Lacey et al., 2010). This hypothesis is consistent with theoretical models predicting that for organisms living in temporally variable environments, the selective benefit of plasticity should depend on the relative frequencies of time during an organism's life when alternative phenotypes have a selective advantage, that is, the frequencies or durations of different selective environments (Gavrilets & Scheiner, 1993a; Gomulkiewicz & Kirkpatrick, 1992; Levins, 1968; Moran, 1992; Van Tienderen & van der Toorn, 1991). Plasticity should be highly favored when the relative frequencies of times favoring alternative phenotypes, for example, in this case, reflective versus nonreflective flowers, are similar. As the frequencies of time favoring the alternative phenotypes deviate from equality, the selective advantage of one phenotype should increase relative to the other, promoting nonplasticity. In an earlier test of these two hypotheses, Lacey et al. (2010) found that the geographic patterns of phenotypic variation in thermal plasticity of floral reflectance were consistent with the Frequency Hypothesis, but not the Magnitude Hypothesis. The new comparisons of the phenotypic data with our molecular genetic data provide additional support for the Frequency Hypothesis by showing that neutral genetic differentiation cannot explain geographic variation in thermal plasticity. Our study has sought to identify past evolutionary mechanisms that have produced the geographic patterns that we see today. Ideally, the next step would be to perform reciprocal transplant experiments to directly test the adaptive hypotheses in today's climate, particularly in light of recent global warming.

Our geographic study differs from many others in terms of geographic scale. Earlier studies have typically examined thermal plasticity of a group of related species over a large geographic range, for example, tropical to temperate regions (e.g., Angilletta, 2009; Ghalambor et al., 2006; Molina‐Montenegro & Naya, 2012). In contrast, we focused on thermal plasticity variation among populations of a single temperate species. When Janzen (1967), who proposed the Magnitude Hypothesis, compared thermal data between tropical versus temperate regions and between lowland versus highland regions in the tropics, he found much greater annual thermal variation in temperate regions and at higher altitudes in the tropics, consistent with the Magnitude Hypothesis. However, his data, and those from other studies (e.g., Molina‐Montenegro & Naya, 2012), also show that monthly temperatures in temperate and highland regions decline substantially more during at least a portion of the year, than in tropical or lowland regions. Therefore, monthly data appear to be consistent also with the Frequency Hypothesis. Because we focused on thermal variation within a single temperate species and limited the time frame to the reproductive season, the relevant time period for reproduction, our ability to test the two hypotheses was greatly improved. We suggest that tests of the two hypotheses at large spatial scales are still needed.

We expect selection on thermal plasticity in nature to be greater than our data suggest. Our *P*_ST_ values are likely conservative underestimates of *Q*_ST_ because we conservatively used a heritability value of 1.0 in our calculations, and reflectance data were collected from clones of the same individuals grown at the same controlled temperatures. Additionally, parental environmental effects had been reduced by passing parents of our experimental plants through one generation in a similar environment, and all individuals were grown in the same environment. Therefore, between‐population differences are due exclusively to genetic factors (see Lacey et al., 2010).

Temperature increases associated with contemporary climate change and local land‐use change, for example, urbanization, are widespread and are already having a strong influence on species distributions (IPCC, 2014; Parmesan, 2006; Visser, 2008; Walther et al., 2002). For example, reproductive seasons are being lengthened, and advances in flowering have been observed in many plant species including *P. lanceolata *(Abu‐Asab, Peterson, Shetler, & Orli, 2001; Cleland, Chuine, Menzel, Mooney, & Schwartz, 2007; Fitter & Fitter, 2002). Phenotypic plasticity has been proposed as a mechanism for coping with climate change because it can provide organisms with the potential to respond rapidly to changes in their environment (Charmantier et al., 2008; Gienapp, Teplitsky, Alho, Mills, & Merilä, 2007; Matesanz, Gianoli, & Valladares, 2010; Przybylo, Sheldon, & Merilä, 2000; Réale, McAdam, Boutin, & Berteaux, 2003; Visser, 2008). However, few empirical studies provide data to test this hypothesis. Given the evidence that thermal plasticity of floral reflectance in *P. lanceolata *is better adapted to environments with short and cool reproductive seasons, our data suggest that the advantage of thermal plasticity will generally decrease in extant populations of other species as well, as warmer weather becomes more prevalent. On the other hand, warming should transform areas beyond today's high latitudinal and altitudinal range limits into suitable habitats. In *P. lanceolata*, floral reflectance plasticity should help facilitate the colonization of new populations toward the poles and higher elevations. Recent ecological niche models are consistent with these predictions (Valladares et al., 2014). Projections suggest the less plastic southern and low‐altitude populations will experience poleward and uphill niche expansion and will retain a large area of suitable habitat over the next few decades (Valladares et al., 2014).

In closing, we emphasize that plastic responses are specific to the traits, environments, and organisms considered and, thus, are as diverse as life itself. As a result, determining whether plasticity, or the evolution of plasticity, can ameliorate the effects of climate change depends on several factors (Munday, Warner, Monro, Pandolfi, & Marshall, 2013; Parmesan, 2006; Visser, 2008; Walther et al., 2002). For example, determining how the environmental sensitivity of different traits functions to influence fitness should help us predict whether or not plasticity will help individuals endure future conditions. In addition, knowledge of the genetic architecture (i.e., the number and effect size of underlying genes) and the inheritance of plasticity should inform our predictions about how traits will evolve under different scenarios. Future studies that examine the relationship between phenotypic plasticity and life history at large spatial scales will improve our understanding of evolutionary processes and thus our ability to predict species persistence in the face of ever changing environmental conditions.

## CONFLICT OF INTEREST

The authors have no conflict of interest to declare.

## AUTHOR CONTRIBUTIONS

L.C.B., D.L.R., and E.P.L conceived the sampling design; L.C.B. conducted laboratory work; and M.M.M, D.L.R., and E.P.L performed analyses and wrote the manuscript.

## Supporting information

FigS1

FigS2

FigS3

## Data Availability

Archival location upon acceptance: https://doi.org/10.5061/dryad.287f2n0. Data to be archived include: (a) AFLP Hickory & STRUCTURE raw input data, population means data, and differentiation matrices files. (b) R scripts for *P*_ST_ 95% CI bootstrapping, environmental PCA, and Mantel tests.
