# Supplementary figures and images for: Natural selection contributes to geographic patterns of thermal plasticity in Plantago lanceolata
Source: Ecol Evol. 2019 Feb 14;9(5):2945–63. doi: 10.1002/ece3.4977 (PMC6405498; doi:10.1002/ece3.4977)

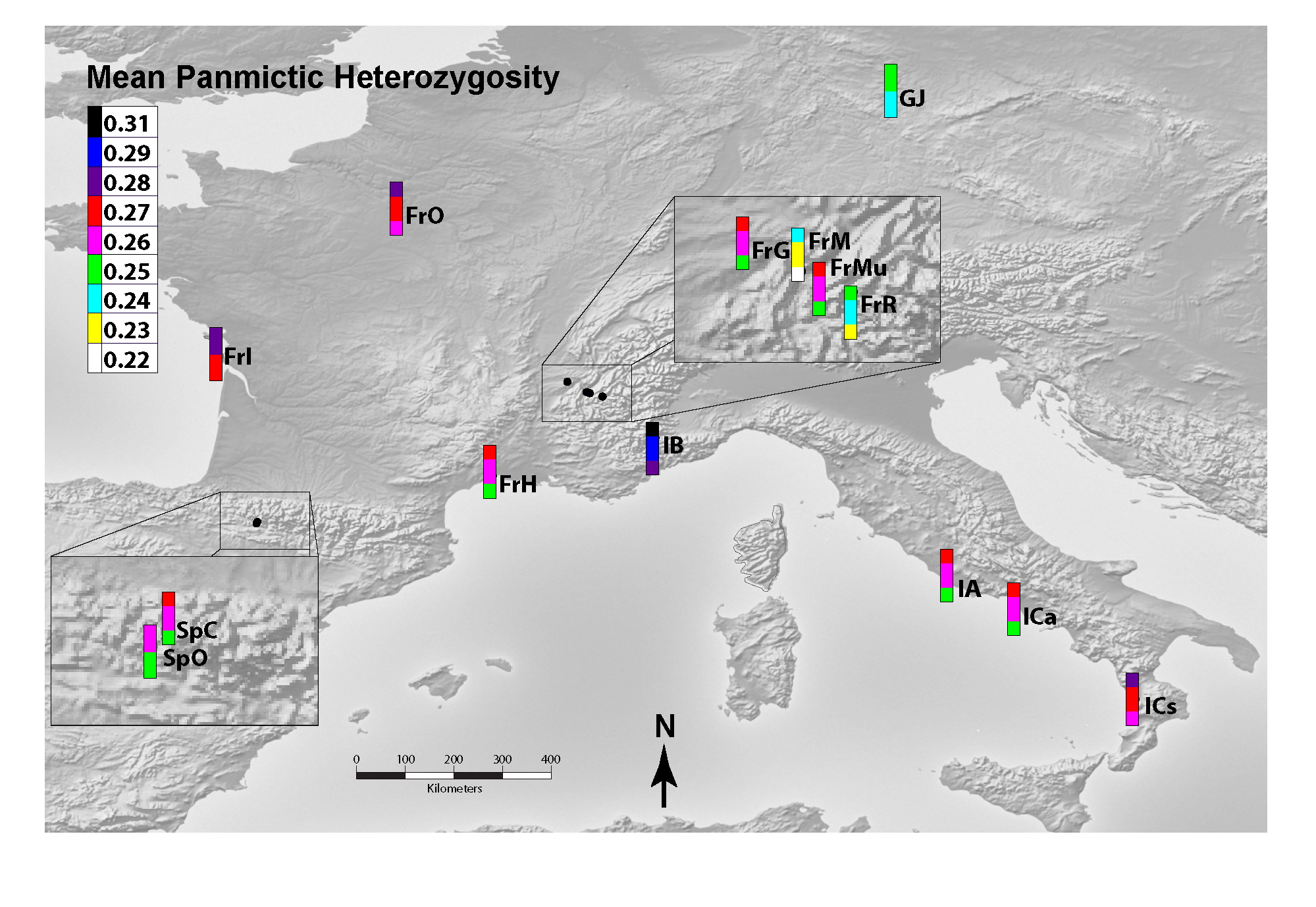

Supplement: Supplementary file 1 — FigS1 [file ECE3-9-2945-s001.tif]

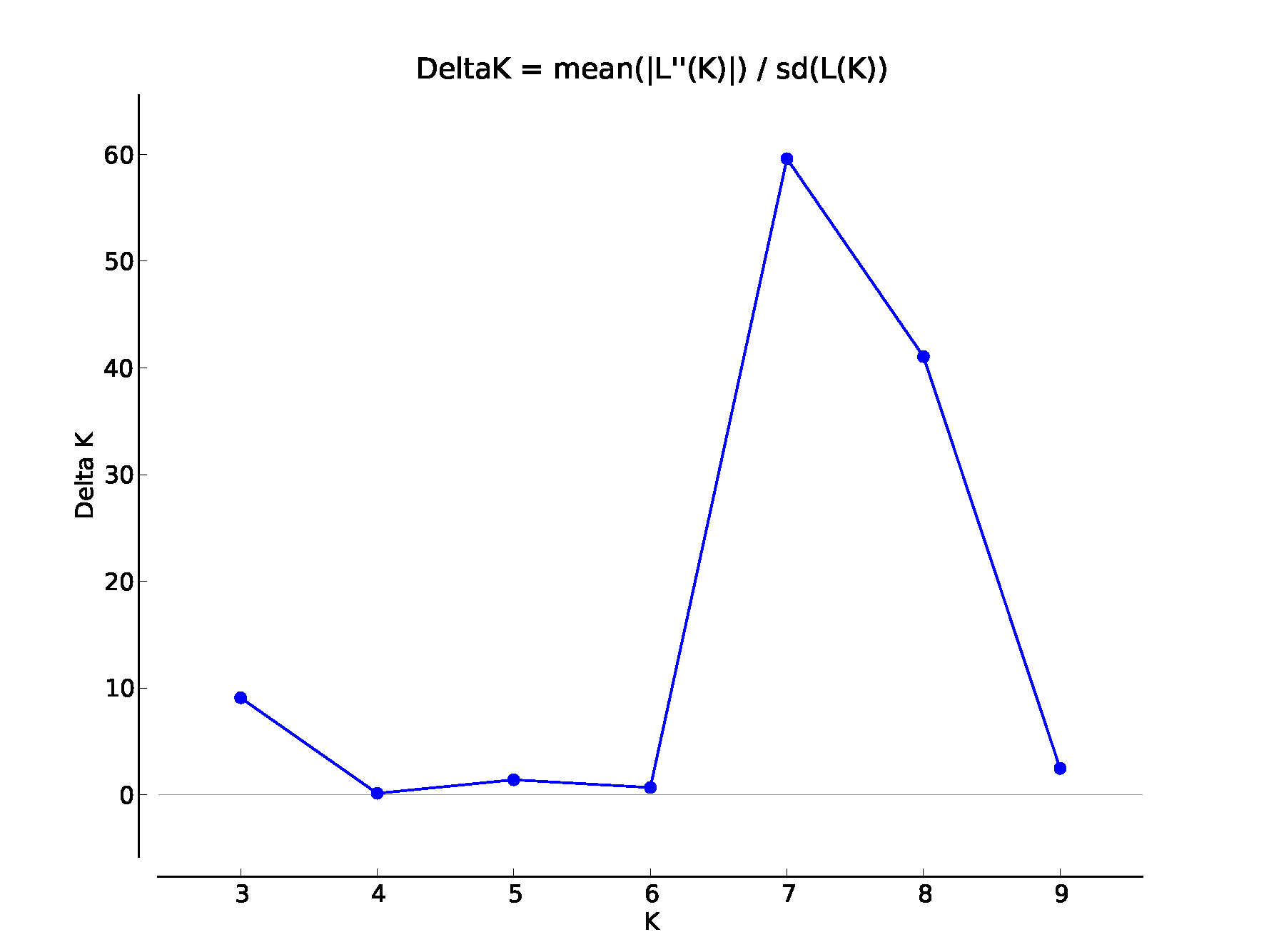

Supplement: Supplementary file 2 — FigS2 [file ECE3-9-2945-s002.tif]

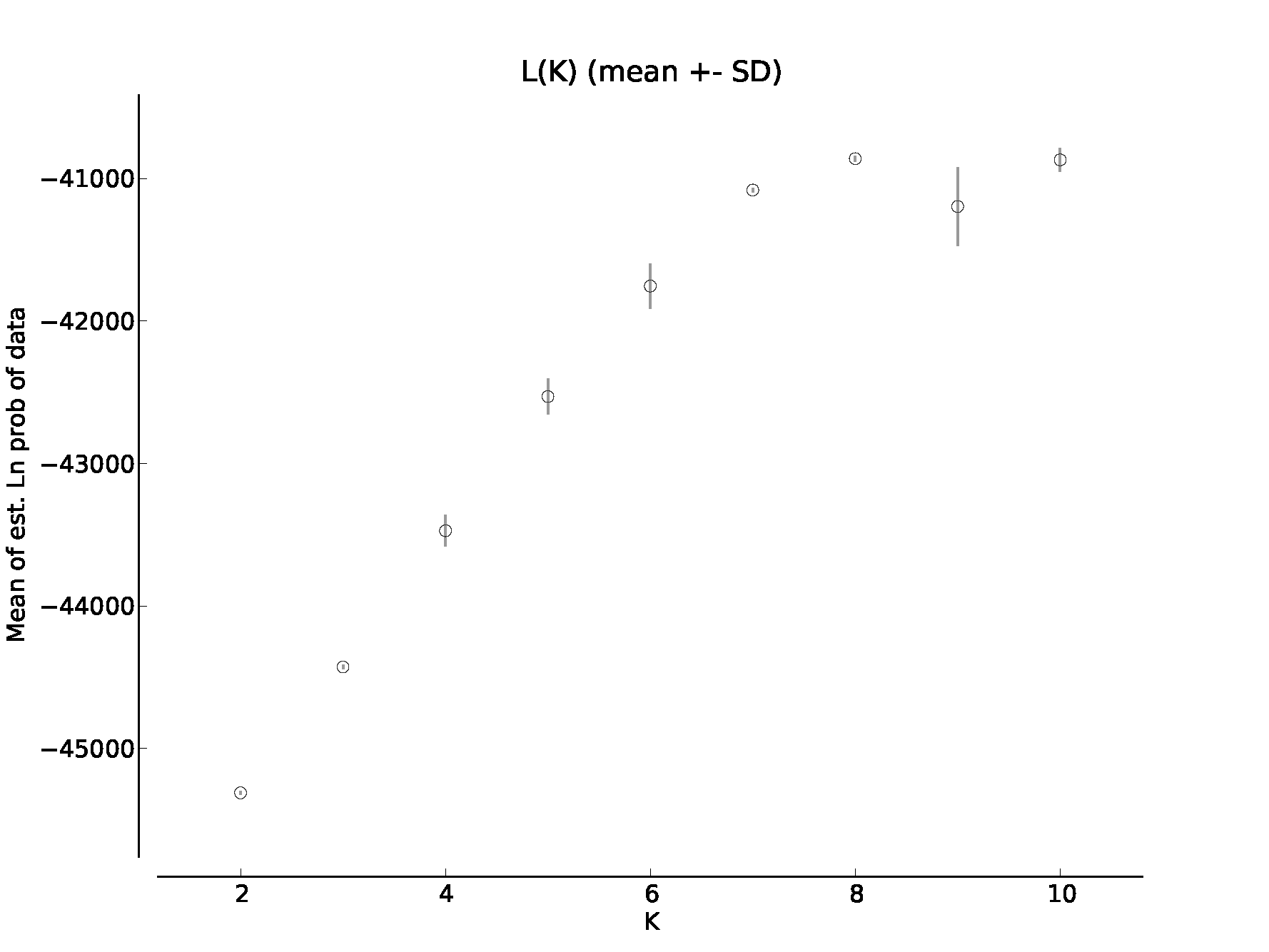

Supplement: Supplementary file 3 — FigS3 [file ECE3-9-2945-s003.tif]
