# Supplementary material for: Natural selection contributes to geographic patterns of thermal plasticity in Plantago lanceolata
Source: Ecol Evol. 2019 Feb 14;9(5):2945–63. doi: 10.1002/ece3.4977 (PMC6405498; doi:10.1002/ece3.4977)
Supplement: Supplementary file 4 [file ECE3-9-2945-s004.docx]

Appendix 1: Supplementary Tables and Figures

Table S1. Scoring error for AFLP markers was determined using individual genotypes repeated within and between FAM and TAMRA (TAM) dyes as the percentage of markers in disagreement.

|  | TAM | FAM | BETWEEN |
| --- | --- | --- | --- |
| **CCAA** |  |  |  |
| Individuals | 5 | 4 | 14 |
| Markers in disagreement | 13 | 9 | 59 |
| Total markers | 226 | 196 | 677 |
| **CCAC** |  |  |  |
| Individuals | 3 | 2 | 0 |
| Markers in disagreement | 5 | 0 | 0 |
| Total markers | 113 | 90 | 0 |
| **CCAG** |  |  |  |
| Individuals | 7 | 2 | 1 |
| Markers in disagreement | 10 | 3 | 2 |
| Total markers | 255 | 74 | 37 |
| **CCAT** |  |  |  |
| Individuals | 7 | 5 | 1 |
| Markers in disagreement | 17 | 6 | 4 |
| Total markers | 189 | 112 | 28 |
| **CCTA** |  |  |  |
| Individuals | 0 | 5 | 0 |
| Markers in disagreement | 0 | 14 | 0 |
| Total markers | 0 | 295 | 0 |
| **CCTC** |  |  |  |
| Individuals | 2 | 5 | 0 |
| Markers in disagreement | 4 | 12 | 0 |
| Total markers | 92 | 209 | 0 |
| **CCTG** |  |  |  |
| Individuals | 0 | 8 | 0 |
| Markers in disagreement | 0 | 16 | 0 |
| Total markers | 0 | 272 | 0 |
| **CCTT** |  |  |  |
| Individuals | 3 | 2 | 0 |
| Markers in disagreement | 5 | 8 | 0 |
| Total markers | 120 | 46 | 0 |
| **Percent Error (±2 SE)** | **5.51±2.29%** | **5.06±1.79%** | **8.86±1.67%** |

Table S2. Regional pairwise genetic differentiation (populations in region) as F_ST_ (2 standard deviations). Populations of *Plantago lanceolata* from southern Europe were grouped into geographic regions based on physical location and inter-regional genetic differentiation was calculated as illustrated in Figure 2. Values were calculated in Hickory using 313 AFLP markers from 315 genotypes. Population symbols identified in Table 1.

| **Region** | **South France** | **West France** | **S.E. France Alps** | **North France** | **Germany** | **North Italy** | **South Italy** | **Spain** |
| --- | --- | --- | --- | --- | --- | --- | --- | --- |
| (Population(s)) | (FrH) | (FrI) | (FrG, FrM, FrMu, FrR) | (FrO) | (GJ) | (IB) | (IA, ICa, ICs) | (SpC, SpO) |
| **South France** | - | - | - | - | - | - | - | - |
| (FrH) |  |  |  |  |  |  |  |  |
| **West France** | 0.149 (0.047) | - | - | - | - | - | - | - |
| (FrI) |  |  |  |  |  |  |  |  |
| **S.E. France Alps** | 0.215 (0.042) | 0.245 (0.043) | - | - | - | - | - | - |
| (FrG, FrM, FrMu, FrR) |  |  |  |  |  |  |  |  |
| **North France** | 0.052 (0.029) | 0.082 (0.039) | 0.186 (0.052) | - | - | - | - | - |
| (FrO) |  |  |  |  |  |  |  |  |
| **Germany** | 0.144 (0.047) | 0.278 (0.064) | 0.273 (0.049) | 0.193 (0.058) | - | - | - | - |
| (GJ) |  |  |  |  |  |  |  |  |
| **North Italy** | 0.107 (0.044) | 0.178 (0.044) | 0.196 (0.05) | 0.122 (0.05) | 0.021 (0.012) | - | - | - |
| (IB) |  |  |  |  |  |  |  |  |
| **South Italy** | 0.107 (0.036) | 0.212 (0.037) | 0.228 (0.044) | 0.164 (0.046) | 0.225 (0.039) | 0.008 (0.006) | - | - |
| (IA, ICa, ICs) |  |  |  |  |  |  |  |  |
| **Spain** | 0.075 (0.023) | 0.195 (0.035) | 0.239 (0.032) | 0.117 (0.035) | 0.129 (0.034) | 0.013 (0.009) | 0.187 (0.031) | - |
| (SpC, SpO) |  |  |  |  |  |  |  |  |

Table S3. Results of multiple regression of distance matrices (MRM) tests of the phenotypic differentiation (P_ST_) in temperature-sensitive floral reflectance plasticity matrix on matrices of geographic distance, genetic differentiation (Jost’s D), and/or environmental differences between *Plantago lanceolata* populations. Environmental variables examined were reproductive season duration (Duration), the proportion of the reproductive season having temperatures less than 15°C (DegMoB15°C), the magnitude of thermal variation during the reproductive season (Magnitude) and total reproductive season precipitation (Precipitation). MRM model r^2^ and regression coefficients obtained from permutation tests are reported. FDR adjusted p-values are listed parenthetically: **bold** = p < 0.05, i*talic* = 0.05 < p < 0.10. See Methods for more information about MRM tests.

| **MRM Model** | **r^2^** | **Intercept** | **Distance** | **Jost's D** | **Duration** | **DegMoB15°C** | **Magnitude** | **Precipitation** |
| --- | --- | --- | --- | --- | --- | --- | --- | --- |
| A. P_ST_ ~ Jost’s D | 0.13 | 3.7E-11 | **-** | 0.365 | **-** | **-** | **-** | **-** |
|  | **(0.006)** | **(0.005)** | **-** | **(0.005)** | **-** | **-** | **-** | **-** |
| B. P_ST_ ~ Jost's D + Distance | 0.18 | 3.17E-11 | 0.294 | 0.177 | **-** | **-** | **-** | **-** |
|  | **(0.006)** | (0.275) | *(0.055)* | (0.189) | **-** | **-** | **-** | **-** |
| C. P_ST_ ~ Jost's D + Duration | 0.15 | 4.08E-11 | **-** | 0.359 | 0.117 | **-** | **-** | **-** |
|  | **(0.006)** | **(0.028)** | **-** | **(0.006)** | (0.227) | **-** | **-** | **-** |
| D. P_ST_ ~ Jost's D + Distance | 0.20 | 3.54E-11 | 0.292 | 0.172 | 0.114 | **-** | **-** | **-** |
| + Duration | **(0.006)** | (0.133) | *(0.057)* | (0.198) | (0.230) | **-** | **-** | **-** |
| E. P_ST_ ~ Jost’s D + DegMoB15°C | 0.17 | 3.23E-11 | **-** | 0.341 | **-** | 0.202 | **-** | **-** |
|  | **(0.006)** | (0.239) | **-** | **(0.007)** | **-** | **(0.046)** | **-** | **-** |
| F. P_ST_ ~ Jost's D + Distance | 0.22 | 2.75E-11 | 0.280 | 0.163 | **-** | 0.191 | **-** | **-** |
| + DegMoB15°C | **(0.006)** | (0.774) | *(0.063)* | (0.217) | **-** | *(0.052)* | **-** | **-** |
| G. P_ST_ ~ Jost's D + Magnitude | 0.16 | 3.88E-11 | **-** | 0.373 | **-** | **-** | 0.154 | **-** |
|  | **(0.006)** | **(0.008)** | **-** | **(0.005)** | **-** | **-** | (0.151) | **-** |
| H. P_ST_ ~ Jost's D + Distance | 0.21 | 3.34E-11 | 0.305 | 0.178 | **-** | **-** | 0.167 | **-** |
| + Magnitude | **(0.006)** | (0.145) | **(0.043)** | (0.182) | **-** | **-** | (0.106) | **-** |
| I. P_ST_ ~ Jost's D + Precipitation | 0.13 | 3.72E-11 | **-** | 0.358 | **-** | **-** | **-** | -0.0239 |
|  | **(0.006)** | **(0.007)** | **-** | **(0.006)** | **-** | **-** | **-** | (0.891) |
| J. P_ST_ ~ Jost’s D + Distance | 0.18 | 3.16E-11 | 0.286 | 0.178 | **-** | **-** | **-** | 0.0112 |
| + Precipitation | **(0.006)** | (0.345) | *(0.054)* | (0.196) | **-** | **-** | **-** | (0.947) |

Table S4. Results of multiple regression of distance matrices (MRM) tests of the phenotypic differentiation (P_ST_) in temperature-sensitive floral reflectance plasticity matrix on matrices of geographic distance, genetic differentiation (Jost’s D), and/or environmental differences between *Plantago lanceolata* populations. Environmental variables examined were principal component axes that combined multiple variables. Thermal_PC combined duration and the proportion of the reproductive season having temperatures less than 15°C. Magnitude_PC combined duration and the magnitude of thermal variation during the reproductive season. Mag_Therm_PC combined duration, proportion of the reproductive season having temperatures less than 15°C, and seasonal magnitude of thermal variation. MRM model r^2^ and regression coefficients obtained from permutation tests are reported. FDR adjusted p-values are listed parenthetically: **bold** = p < 0.05, i*talic* = 0.05 < p < 0.10. See Methods for more information about MRM tests.

| **MRM Model** | **r^2^** | **Intercept** | **Distance** | **Jost's D** | **Thermal_PC** | **Magnitude_PC** | **Mag_Therm_PC** |
| --- | --- | --- | --- | --- | --- | --- | --- |
| A. P_ST_ ~ Jost's D + Thermal_PC | 0.18 | 3.41E-11 | **-** | 0.326 | 0.224 | **-** | **-** |
|  | **(0.006)** | **(0.032)** | **-** | **(0.008)** | **(0.040)** | **-** | **-** |
| B. P_ST_ ~ Jost's D + Distance | 0.23 | 2.93E-11 | 0.273 | 0.153 | 0.209 | **-** | **-** |
| + Thermal_PC | **(0.006)** | (0.521) | *(0.070)* | (0.242) | *(0.051)* | **-** | **-** |
| C. P_ST_ ~ Jost's D + Magnitude_PC | 0.16 | 4.71E-11 | **-** | 0.366 | **-** | 0.153 | **-** |
|  | **(0.006)** | **(0.045)** | **-** | **(0.005)** | **-** | (0.143) | **-** |
| D. P_ST_ ~ Jost's D + Distance | 0.21 | 4.17E-11 | 0.293 | 0.179 | **-** | 0.152 | **-** |
| + Magnitude_PC | **(0.006)** | *(0.095)* | *(0.055)* | (0.180) | **-** | (0.138) | **-** |
| E. P_ST_ ~ Jost’s D + Mag_Therm_PC | 0.18 | 3.18E-11 | **-** | 0.341 | **-** | **-** | 0.221 |
|  | **(0.006)** | (0.305) | **-** | **(0.007)** | **-** | **-** | **(0.050)** |
| F. P_ST_ ~ Jost's D + Distance | 0.23 | 2.7E-11 | 0.280 | 0.163 | **-** | **-** | 0.211 |
| + Mag_Therm_PC | **(0.006)** | (0.791) | *(0.064)* | (0.215) | **-** | **-** | *(0.059)* |

Table S5. Mantel correlations between geographic distance, neutral genetic differentiation (F_ST_ and Jost’s D) and pairwise differences among environmental properties of the reproductive season. Environmental variables examined were reproductive season duration (Duration), the proportion of the reproductive season under 15°C (DegMoB15°C), the magnitude of thermal variation of the reproductive season (Magnitude), total reproductive season precipitation (Precipitation), and three principal components axes integrating these variables (Thermal_PC, Magnitude_PC, Mag_Therm_PC, see text for details). Mantel correlation coefficients and p-values were obtained from permutation tests. FDR-adjusted p-values are reported.

|  |  |  | r | p |
| --- | --- | --- | --- | --- |
| Distance | ~ | Duration | 0.04 | 0.654 |
| F_ST_ | ~ | Duration | 0.15 | 0.393 |
| Jost's D | ~ | Duration | 0.05 | 0.654 |
| Distance | ~ | DegMoB15°C | 0.12 | 0.468 |
| F_ST_ | ~ | DegMoB15°C | 0.20 | 0.393 |
| Jost's D | ~ | DegMoB15°C | 0.12 | 0.468 |
| Distance | ~ | Magnitude | -0.07 | 0.654 |
| F_ST_ | ~ | Magnitude | 0.00 | 0.807 |
| Jost's D | ~ | Magnitude | -0.05 | 0.654 |
| Distance | ~ | Precipitation | -0.28 | 0.393 |
| F_ST_ | ~ | Precipitation | -0.17 | 0.541 |
| Jost's D | ~ | Precipitation | -0.27 | 0.393 |
| Distance | ~ | Thermal_PC | 0.17 | 0.393 |
| F_ST_ | ~ | Thermal_PC | 0.29 | 0.393 |
| Jost's D | ~ | Thermal_PC | 0.17 | 0.393 |
| Distance | ~ | Magnitude_PC | 0.00 | 0.807 |
| F_ST_ | ~ | Magnitude_PC | 0.07 | 0.654 |
| Jost's D | ~ | Magnitude_PC | 0.17 | 0.393 |
| Distance | ~ | Mag_Therm_PC | 0.11 | 0.545 |
| F_ST_ | ~ | Mag_Therm_PC | 0.20 | 0.393 |
| Jost's D | ~ | Mag_Therm_PC | 0.11 | 0.541 |
|  |  |  |  |  |
|  |  |  |  |  |
|  |  |  |  |  |

Table S6. Runs test p-values from linear regressions of phenotypic differentiation (P_ST_) in temperature-sensitive floral reflectance plasticity and neutral genetic differentiation (F_ST_ and Jost’s D) on an x-axis of (A) increasing geographic distance and (B-H) environmental differences. A runs test p>0.05 indicates no deviation from linearity.

|  | P_ST_ | F_ST_ | Jost's D |
| --- | --- | --- | --- |
| A. Geographic Distance | 0.158 | 0.523 | 0.664 |
| B. Reproductive Season Duration | **0.009** | **0.03** | *0.07* |
| C. Proportion of Reproductive Season Under 15°C | 0.981 | 0.207 | 0.956 |
| D. Magnitude of Thermal Variation In Reproductive Season | 0.274 | 0.685 | 0.146 |
| E. Thermal_PC | 0.661 | 0.2 | 0.225 |
| F. Magnitude_PC | 0.604 | 0.9 | 0.736 |
| G. Mag_Therm_PC | 0.254 | 0.42 | 0.746 |
| H. Total Precipitation in Reproductive Season | 0.127 | 0.861 | 0.512 |

Figure S1. Mean panmictic heterozygosity (center of color bar) ± 95% CI (upper and lower portion of color bar) of 14 *Plantago lanceolata* populations from southern Europe. Values were calculated in Hickory using 313 AFLP markers from 315 genotypes. Locations are approximate, symbols as in Table 1. Pop-out boxes zoomed 4x.

Figure S2. Delta K of STRUCTURE runs from K=2-10 suggest AFLP data from 14 *Plantago lanceolata* populations from southern Europe best fit into 7 Groups. Runs were combined and interpreted with Structure Harvester (Earl, 2012), using the methods of Evanno *et al.* (2005).

Figure S3. Mean (± SD) of estimated Ln probability of data from STRUCTURE runs from K=2-10 suggest AFLP data from 14 *Plantago lanceolata* populations from southern Europe best fit into 7 or 8 groups. Runs were combined and interpreted with Structure Harvester (Earl, 2012), using the methods of Pritchard *et al.* (2000).
